# Serum concentrations of levosimendan and its metabolites OR-1855 and OR-1896 in cardiac surgery patients with cardiopulmonary bypass

**DOI:** 10.3389/fcvm.2024.1406338

**Published:** 2024-08-08

**Authors:** Hannah Kipka, Uwe Liebchen, Max Hübner, Georg Höfner, Otto Frey, Klaus T. Wanner, Erich Kilger, Christian Hagl, Roland Tomasi, Hanna Mannell

**Affiliations:** ^1^ Doctoral Program Clinical Pharmacy, LMU University Hospital, LMU Munich, Germany; ^2^ Institute of Cardiovascular Physiology and Pathophysiology, Biomedical Center, LMU Munich, Planegg, Germany; ^3^ Department of Anaesthesiology, LMU University Hospital, LMU Munich, Munich, Germany; ^4^ Walter Brendel Center of Experimental Medicine, LMU Munich, LMU University Hospital, Munich, Germany; ^5^ Department of Pharmacy, Center for Drug Research, Ludwig-Maximilians-Universität München, Munich, Germany; ^6^ Department of Pharmacy, General Hospital of Heidenheim, Heidenheim, Germany; ^7^ Department of Cardiac Surgery, LMU University Hospital, LMU Munich, Germany; ^8^ DZHK (German Centre of Cardiovascular Research), Partner Site Munich Heart Alliance, Munich, Germany; ^9^ Physiology, Institute for Theoretical Medicine, Faculty of Medicine, University of Augsburg, Augsburg, Germany

**Keywords:** levosimendan, OR-1896, OR-1855, cardiac insufficiency, cardiopulmonary bypass, serum levels, cardiac surgery

## Abstract

**Background:**

The inotropic drug levosimendan is often used as an individualized therapeutic approach perioperatively in cardiac surgery patients with cardiopulmonary bypass (CPB). Data regarding serum concentrations of levosimendan and its metabolites within this context is lacking.

**Methods:**

In this retrospective descriptive proof-of-concept study, total serum concentrations (TSC) and unbound fractions (UF) of levosimendan and its metabolites OR-1896 and OR-1855 in cardiac surgery patients with CPB were measured using LC-ESI-MS/MS. Simulation of expected levosimendan TSC was performed using Pharkin 4.0. Serum NT-proBNP was assessed with ELISA.

**Results:**

After levosimendan infusion (1.25 mg or 2.5 mg, respectively) after anaesthesia induction, a median TSC of 1.9 ng/ml and 10.4 ng/ml was determined in samples taken directly after surgery (T1). Median TSC of 7.6 ng/ml and 22.0 ng/ml, respectively, were simulated at T1. Whereas 1.1 ng/ml and 1.6 ng/ml TSC of OR-1896, respectively, was quantified the day after surgery (T2), TSC of the intermediate metabolite OR-1855 was mostly below the lower limit of quantification (LLOQ). The UF was 0.5% and 1.1% for levosimendan and 64.1% and 52.1% for OR-1896, respectively, with over half the samples being below LLOQ. NT-proBNP concentrations before surgery and T2 did not differ.

**Discussion:**

The low TSC, UF and unchanged NT-proBNP levels in combination with high variation of serum levels between patients suggest a need for optimized dosing regimen of levosimendan combined with therapeutic drug monitoring for such an individualized approach. In addition, the differences between the measured and estimated concentrations may suggest a possible influence of CPB on levosimendan serum concentrations.

## 1 Introduction

Perioperative left ventricular dysfunction is a major complication after cardiac surgery and is associated with increased mortality (1). Catecholamines and phosphodiesterase type 3 inhibitors (2) are the therapy of choice for postoperative hemodynamic support, but meta-analyses and observational studies suggest increased mortality when using these agents (3, 4). An alternative inotropic drug therapy for ventricular dysfunction could be the perioperatively administration of levosimendan (Simdax, Orion Pharma). As a calcium sensitizer, levosimendan has positive inotropic effects and a network meta-analysis showed it to be the most likely inotrope to reduce mortality among patients undergoing cardiac surgery (5). In addition to the improvement of cardiac output with minimal effect on myocardial oxygen consumption (6), it has antioxidative, anti-inflammatory, and direct cardioprotective effects (6). 4%–7% of the administered dose is metabolized by intestinal bacteria to the metabolite OR-1855, which is then acetylated by hepatic N-acetyltransferase (NAT2) to the pharmacologically active OR-1896 (7). Whereas levosimendan has a short half-life of 1 h, the half-life of the metabolites is 70–80 h and it is therefore hypothesized that the long-term effects arise from the metabolites (8), of which OR-1896 inhibits myocardial PDE3 (9). Interestingly, despite the positive effects initially shown in meta-analyses (10–12), three randomized controlled trials comparing levosimendan with placebo on top of standard care in cardiac surgical patients failed to show effectiveness as a prophylactic treatment for the prevention of postoperative low cardiac output (LCO) in patients with impaired left ventricular ejection fraction (EF) (13, 14), and in reducing mortality in patients developing post-bypass LCO syndrome (15).

In our cardiac surgery center, patients with impaired EF undergoing cardiac surgery receive a patient specific and thus individualized levosimendan dose tailored to their specific needs to achieve hemodynamic stabilization (16). Accordingly, to reduce adverse events and to prevent LCO syndrome, a single levosimendan dose of 1.25 mg and up to 2.5 mg is administered with a flow rate between 0.2 and 0.25 μg kg^–1^ min^–1^ intra- and perioperatively based on the studies from Tritapepe et al. (17, 18). A retrospective study evaluating this individualized approach showed positive clinical effects, such as reduced use of catecholamines, renal replacement techniques and mortality compared to published studies using higher non-invidualized levosimendan application (16). However, the observed improved clinical effects of the individualized treatment regimen were not statistically confirmed. Moreover, as this individualized treatment regimen uses much lower total levosimendan dosages than most other studies, it is of high interest to investigate the serum levels of levosimendan in these patients to confirm the observed positive effects. Especially since cardiopulmonary bypass (CPB) during surgery can have negative effects on the pharmacokinetics of drugs (19, 20). So far, serum concentrations of levosimendan and its metabolites in these patients are not available. Moreover, real-life data regarding serum concentrations of levosimendan and its metabolites within cardiac surgery patients upon CPB procedure is lacking in general. Therefore, the aim of this descriptive investigation was to determine the serum levels of levosimendan, OR-1855 and OR-1896 in this patient population.

For this, we recently developed and validated a method for therapeutic drug monitoring (TDM) of levosimendan, OR-1855 and OR-1896 (21), enabling rapid and simultaneous detection of these compounds in serum (21). In the current descriptive study, we thus first measured total serum concentrations (TSC) of levosimendan and its two metabolites OR-1855 and OR-1896 retrospectively in patients receiving different individualized dosing regimens of levosimendan perioperatively, according to Woehrle et al. (16), and compared these with simulated serum concentrations. Next, we detected the unbound fractions, which are the therapeutic effective fractions, of levosimendan, OR-1896 and OR-1855. Finally, we measured levels of NT-proBNP, a cardiac biomarker, before and after levosimendan administration to objectively investigate a possible benefit of the measured concentrations.

## 2 Material and methods

### 2.1 Chemicals

^13^C_6_ labelled internal standards of levosimendan, OR-1896, and OR-1855 were obtained from Orion Pharma (Espoo, Finland).

### 2.2 Human samples

Pseudonymized serum supernatants from the local biobank of the department of Anaesthesiology were screened for selection of samples from patients within the study cohort of Woehrle et al. (16), who underwent elective cardiac surgery with CPB at the department of cardiac surgery at the LMU University hospital and who received levosimendan within the perioperative management according to the individualized therapeutic regimen (16). Samples not matching this description were not considered. Samples taken shortly after induction of anaesthesia and before levosimendan infusion (T0), at the end of surgery (T1) and the first day after surgery (T2) were available and stored at −80°C. Available in the local biobank were samples from 18 patients, who received a single total levosimendan dose of 1.25 mg or 2.5 mg (starting with 1.25 mg and increasing the dose to 2.5 mg) shortly after induction of anaesthesia as well as two levosimendan doses of 1.25 mg or 2.5 mg as the first dose shortly after induction of anaesthesia and the second dose of 1.25 mg or 2.5 mg, respectively, within the first post-operative day with a flow rate between 0.2 and 0.25 μg kg^–1^ min^–1^, according to their specific needs to reach hemodynamic stability as described by Woehrle et al. (16). After anonymization, levosimendan and its metabolites were retrospectively measured in the supernatants. According to in-house guidelines, patients with EF < 25% undergoing CABG, patients with EF < 40% along with GFR < 60 ml/min undergoing CABG or involved in combined procedures, are treated with levosimendan perioperatively. Anaesthesia induction is performed as previously described (16). The CPB circuit is primed with 1000 ml of a balanced electrolyte solution (Jonosteril) and 10,000 IE heparin. The investigation was approved by the clinical ethics committee at the medical faculty, LMU Munich (identification code 17–241; 20–1089).

### 2.3 Sample preparation & LC-ESI-MS/MS

Serum levels of levosimendan, OR-1855 and OR-1896 were quantified with a FDA validated LC-ESI-MS/MS protocol as previously described with lower limits of quantification (LLOQ) for levosimendan of 0.126 ng/ml, OR-1855 of 0.305 ng/ml and OR-1896 of 0.368 ng/ml (21). For unbound fractions (UF), 500 µl of serum was centrifuged in Centrifree® tubes (Merck, Germany) for 30 min at 2,000 × g with a 34° fixed-angle rotor. Liquid-liquid extraction was performed as previously described (21).

### 2.4 Albumin BCG assay

Serum albumin was measured with the BCG Assay Kit (Sigma Aldrich, Germany) according to supplier's protocol.

### 2.5 NT-proBNP measurements

NT-proBNP levels in patientś serum samples were measured with the Human NT-proBNP Elisa Kit (Kelowna BC, Canada) according to supplieŕs protocol.

### 2.6 Simulation of levosimendan serum levels in cardiac surgery patients

The expected serum levels of levosimendan were visualised with Pharkin 4.0 (Heidenheim, Germany). Body weight, height, age, and gender as well as the dose (mg) with the exact dosing intervals and infusion length were included in the calculation. A distribution volume (Vd) of 0.2 L/kg and a half-life of 1.0 h (22) were specified for simulation. The expected concentrations were estimated assuming first-order linear kinetics and a one-compartment model.

### 2.7 Statistical analysis

Statistical analysis was performed using Sigma Plot 12.0 (Systat Software, Inpixon, Düsseldorf, Germany). Data are presented as mean ± SEM or median with range. For comparison of more than two datasets, one-way analysis of variance (ANOVA) was used. For comparison of correlated samples without normal distribution, the Wilcoxon signed rank test was used. Differences were considered significant at *p* < 0.05.

## 3 Results

### 3.1 Study population

Total serum concentrations (TSC) of levosimendan, OR-1855 and OR-1896 were measured retrospectively in samples from 18 cardiac surgery patients with CPB, receiving levosimendan in the perioperative setting (for details, see materials and methods). Patient characteristics are summarized in Table 1. Five patients received 1.25 mg levosimendan after induction of anaesthesia, whereas two patients received an additional second dose of 1.25 mg levosimendan after surgery. Furthermore, six patients received 2.5 mg of levosimendan after induction of anaesthesia and five patients received a second additional dose of 2.5 mg levosimendan after surgery (Supplementary Table S1).

**Table 1:** Patient characteristics.

	Levosimendan 1.25 mg (*n* = 7)	Levosimendan 2.50 mg (*n* = 11)
Gender, f/m (%)	1/7 (12.5%/87.5%)	3/9 (25%/75%)
Age, years (range)	66 (50–79)	67 (54–86)
BMI, kg/m^2^ (±SEM)	25.8 (±2.3)	27.6 (±3.2)
eGFR, ml/min/1.73 m^2^ (±SEM)	77.0 (±5.1)	79.2 (±9.8)
CPB duration, hours (±SEM)	2.4 (±0.5)	2.0 (±0.7)
CPB haemofiltration, *n* (%)	4 (57%)	4 (33.3%)

F, female; m, male; CPB, cardiopulmonary bypass;

For calculation of estimated glomerular filtration rate (eGFR), the CKD-EPI formula was used.

### 3.2 Total serum concentrations of levosimendan and its metabolites OR-1855 & OR-1896 in cardiac surgery patients with CPB

**Figure 1:**
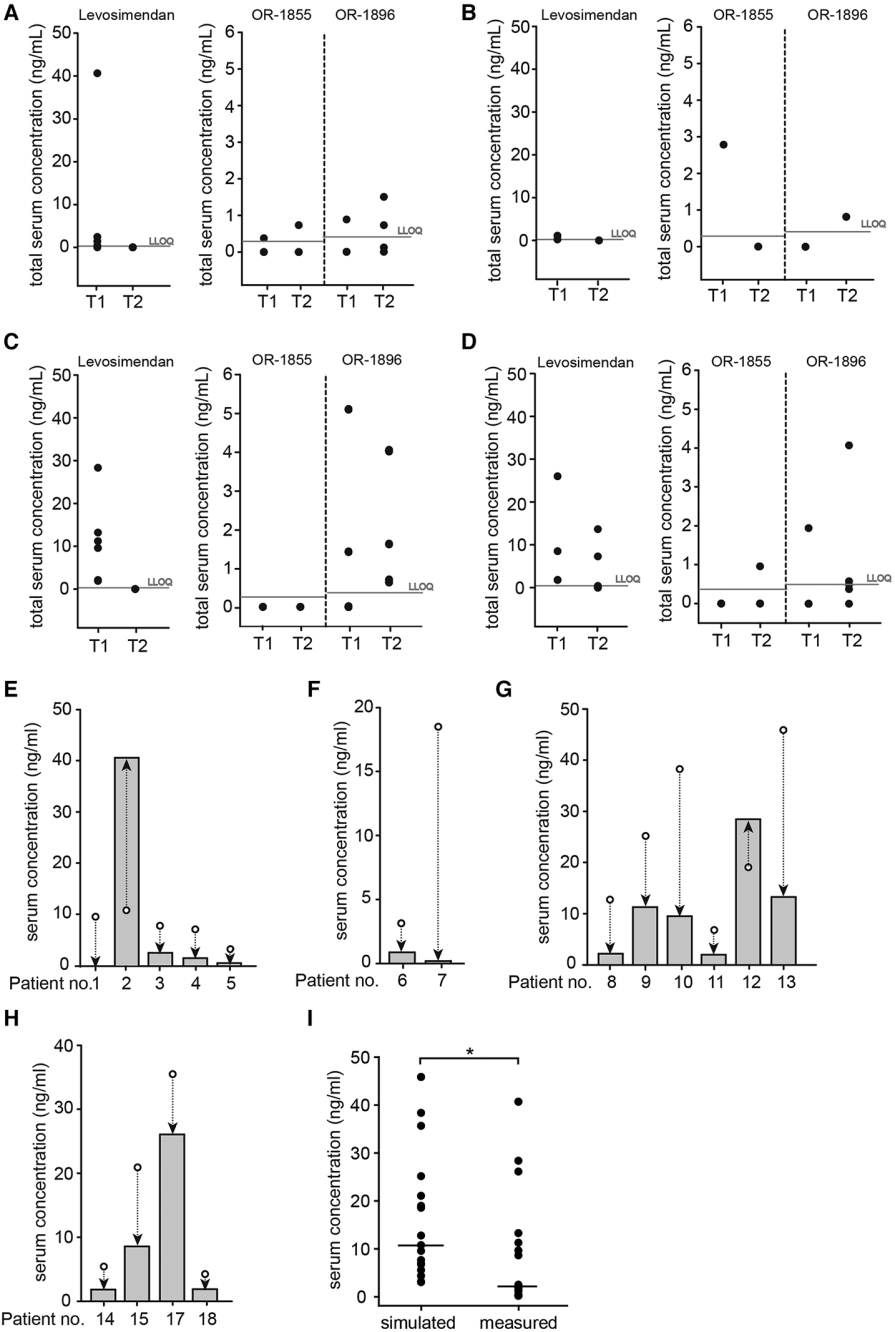
TSC of levosimendan and metabolites in cardiac surgery patients with CPB and comparison of simulated and measured serum levels of levosimendan for each patient at T1. Total serum concentrations (TSC, ng/ml) were measured in samples from cardiac surgery patients with CPB receiving levosimendan after induction of anaesthesia at dosages of (**A**) 1.25 mg of levosimendan (levosimendan *n* = 5; OR-1896 *n* = 4 and OR-1855 *n* = 4); (**B**) 1.25 mg of levosimendan as well as a second dose of 1.25 mg 3–4 h after surgery (*n* = 2); (**C**) 2.5 mg of levosimendan (*n* = 6); (**D**) 2.5 mg of levosimendan as well as a second dose of 2.5 mg 5–21 h after surgery (*n* = 4). T1: Shortly after cardiac surgery; T2: First day after cardiac surgery; grey horizontal line: lower limit of quantification (LLOQ; levosimendan 0.126 ng/ml; OR-1855 0.305 ng/ml; OR-1896 0.368 ng/ml) (21). (**E**) Comparison of measured levosimendan TSC (grey bars) and estimated TSC (white circles) at T1 in patients receiving 1.25 mg of levosimendan (*n* = 5); (**F**) Comparison of measured levosimendan TSC (grey bars) and estimated TSC (white circles) at T1 in patients receiving 1.25 mg of levosimendan as well as a second dose of 1.25 mg 3–4 h after surgery (*n* = 2); (**G**) Comparison of measured levosimendan TSC (grey bars) and estimated TSC (white circles) at T1 in patients receiving 2.5 mg of levosimendan (*n* = 6); (**H**) Comparison of measured levosimendan TSC (grey bars) and estimated TSC (white circles) at T1 in patients receiving 2.5 mg of levosimendan as well as a second dose of 2.5 mg 5–21 h after surgery (*n* = 4). Serum concentrations (ng/ml) were calculated using individual patient data. The simulation for patient 16 could not be performed due to insufficient serum volume for quantification at T1. Arrows show difference between simulated and measured serum concentrations. (**I**) Comparison of simulated levosimendan TSC with measured TSC within the total patient population at T1 (*n* = 17; *p < 0.05 Wilcoxon signed rank test). Data are expressed as dot plots with the respective median (black line).

Simultaneous measurement of levosimendan, OR-1855 and OR-1896 in serum showed that patients receiving a single (*n* = 5) or double (*n* = 2) infusion with 1.25 mg levosimendan had low levosimendan serum concentrations [1.9 ng/ml (0.4–40.6 ng/ml); *n* = 4; bLLOQ *n* = 1 and 0.67 ng/ml (0.2–1.1 ng/ml; *n* = 2) at T1, respectively; Figures 1 A,B]. In contrast, we detected higher serum concentrations in samples from patients receiving a single dose of 2.5 mg levosimendan (*n* = 6) shortly after induction of anaesthesia [10.4 ng/ml (1.9–28.3 ng/ml) at T1; Figure 1C]. In case of an additional infusion of 2.5 mg levosimendan after surgery (*n* = 5), levosimendan was detectable at T1 (5.2 ng/ml (1.8–26.1 ng/ml) and even at T2 [3.9 ng/ml (0.15–13.7 ng/ml); *n* = 4; bLLOQ *n* = 1; Figure 1D]. Regarding the metabolites, concentrations of OR-1896 were quantified at T2 upon 1.25 mg levosimendan [1.1 ng/ml (0.73–1.5 ng/ml); *n* = 2; bLLOQ *n* = 2] as well as upon 2.5 mg levosimendan (1.6 ng/ml [0.6–4.0 ng/ml, (*n* = 5)] (Figures 1A–D). Interestingly, in 87% of the samples OR-1855 levels were below the LLOQ. High variation of levosimendan and OR-1896 serum levels were observed between patients. TSC in all individual patients are listed in Supplementary Table S1.

### 3.3 Measurement of the unbound fraction of levosimendan, OR-1855 & OR-1896 in cardiac surgery patients with CPB

As levosimendan has high binding affinity to serum albumin (97%–98%) (23) and thus only a small amount is unbound and therapeutically effective, we next detected the unbound fraction (UF). For the dose of 1.25 mg levosimendan, the UF of levosimendan was only detected in one patient sample, as the rest had TSC values below LLOQ. The measured UF of levosimendan after application of 2.5 mg in a single or double infusion were 1.1% (0.6–1.1%; *n* = 3) and 0.5% (*n* = 1), respectively (Table 2). In contrast to levosimendan (UF 1%–2%), approximately 60% of the metabolites OR-1855 and OR-1896 are found as unbound fractions (24). For the 1.25 mg levosimendan dose, we detected an UF of 44.0% (*n* = 1) for OR-1855 and 64.1% (*n* = 1; bLLOQ *n* = 1, Table 2) for OR-1896, respectively, whereby two samples had a TSC below LLOQ. In case of a double infusion (1.25 mg), the UF of OR-1896 was 54.1% (*n* = 1, Table 2). At a dose of 2.5 mg levosimendan, no UF of OR-1855 could be quantified neither for a single nor for a double infusion (Table 2). However, we detected an UF of 52.1% (44.8–68.9%; *n* = 5) and 49.4% (*n* = 1) for OR-1896 (Table 2). As we detected lower unbound fractions of levosimendan and OR-1896, and because surgeries may also alter albumin concentrations, the respective albumin concentrations in the serum samples were measured. As seen in Table 2, these were within the normal range (4.1 g/dl (2.2–5.8 g/dl) at T1; 4.4 g/dl (2.8–5-6 g/dl) at T2).

**Table 2:** TSC & UF of levosimendan and metabolites.

Applic. during surgery	TSC (ng/ml)	UF (%)	Albumin (g/dl)
Levosimendan 1.25 mg (*n* = 5)	1.9 (0.4–40.6; *n* = 4) bLLOQ (*n* = 1)	0.5*	4.2 (4.2–4.3)
OR-1855 (*n* = 4)	0.73 (*n* = 1) bLLOQ (*n* = 3)	44.0*	4.0 (2.8–5.0)
OR-1896 (*n* = 4)	1.1 (0.73–1.5; *n* = 2) bLLOQ (*n* = 2)	64.1▴ bLLOQ	4.0 (2.8–5.0)
Levosimendan 2.50 mg (*n* = 6)	10.4 (1.9–28.3)	1.1 (0.6–1.1)•	3.3 (2.6–5.0)
OR-1855 (*n* = 5)	bLLOQ	bLLOQ	3.8 (3.1–5.6)
OR-1896 (*n* = 5)	1.6 (0.6–4.0)	52.1 (44.8–68.9)	3.8 (3.1–5.6)
Applic. during & after surgery	TSC (ng/ml)	UF (%)	Albumin (g/dl)
Levosimendan 1.25 mg (*n* = 2)	0.67 (0.21–1.1)	bLLOQ	4.5 (4.1–4.8)
OR-1855 (*n* = 1)	bLLOQ	bLLOQ	4.6
OR-1896 (*n* = 1)	0.8	54.1	4.6
Levosimendan 2.5 mg (*n* = 4)	5.2 (1.8–26.1)	0.5*	3.8 (2.2–5.8)
OR-1855 (*n* = 5)	0.96 (*n* = 1) bLLOQ (*n* = 4)	i.s.v	4.7 (4.1–4.9)
OR-1896 (*n* = 5)	0.60 (0.44–4.1; *n* = 4) bLLOQ (*n* = 1)	49.4*, i.s.v	4.7 (4.1–4.9)

bLLOQ, below lower limit of quantification; TSC, total serum concentration; UF, unbound fraction; levosimendan at T1; OR-1,855 & OR-1,896 at T2. *n = 1; ▴ *n* = 2; • *n* = 3; i.s.v: insufficient serum volume; Data is expressed as median with the respective (range). No statistical difference in TSC nor in albumin concentrations between the treatment groups was found (both parameters were tested with one-way ANOVA on ranks; groups with less than three samples were not included in the statistical analyses).

### 3.4 Simulated serum levels of levosimendan in cardiac surgery patients

To be able to estimate if the CPB procedure has an influence on levosimendan and metabolite serum levels in these patients, we used individual patient data to simulate the expected levosimendan serum levels after the respective applied doses. For a dose of 1.25 mg levosimendan, the median TSC at T1 was indicated with 7.6 ng/ml [(3.0–10.7 ng/ml); *n* = 5, Figure 1E]. In this group, lower levosimendan TSC were measured for all patients but one in comparison to the simulated concentrations. Patients who received a double infusion of 1.25 mg levosimendan showed a simulated median serum level of 10.8 ng/ml (3.1–18.5 ng/ml; *n* = 2, Figure 1F) at T1, which was higher than the actual measured TSC. Median serum concentrations of 22.0 ng/ml (6.7–45.8 ng/ml) and 13.3 ng/ml (4.3–35.6 ng/ml) at T1 were simulated in patients with a single (*n* = 6) or double (*n* = 4) infusion of 2.5 mg levosimendan, respectively. As seen in Figures 1G,H, the measured TSC were markedly lower than the simulated TSC for 9 of 10 patients. Within the total patient population, regardless of applied levosimendan dose, the measured TSC were significantly lower than the simulated TSC (*p* < 0.05; Figure 1I).

### 3.5 NT-proBNP concentrations in samples from cardiac surgery patients with CPB

**Figure 2:**
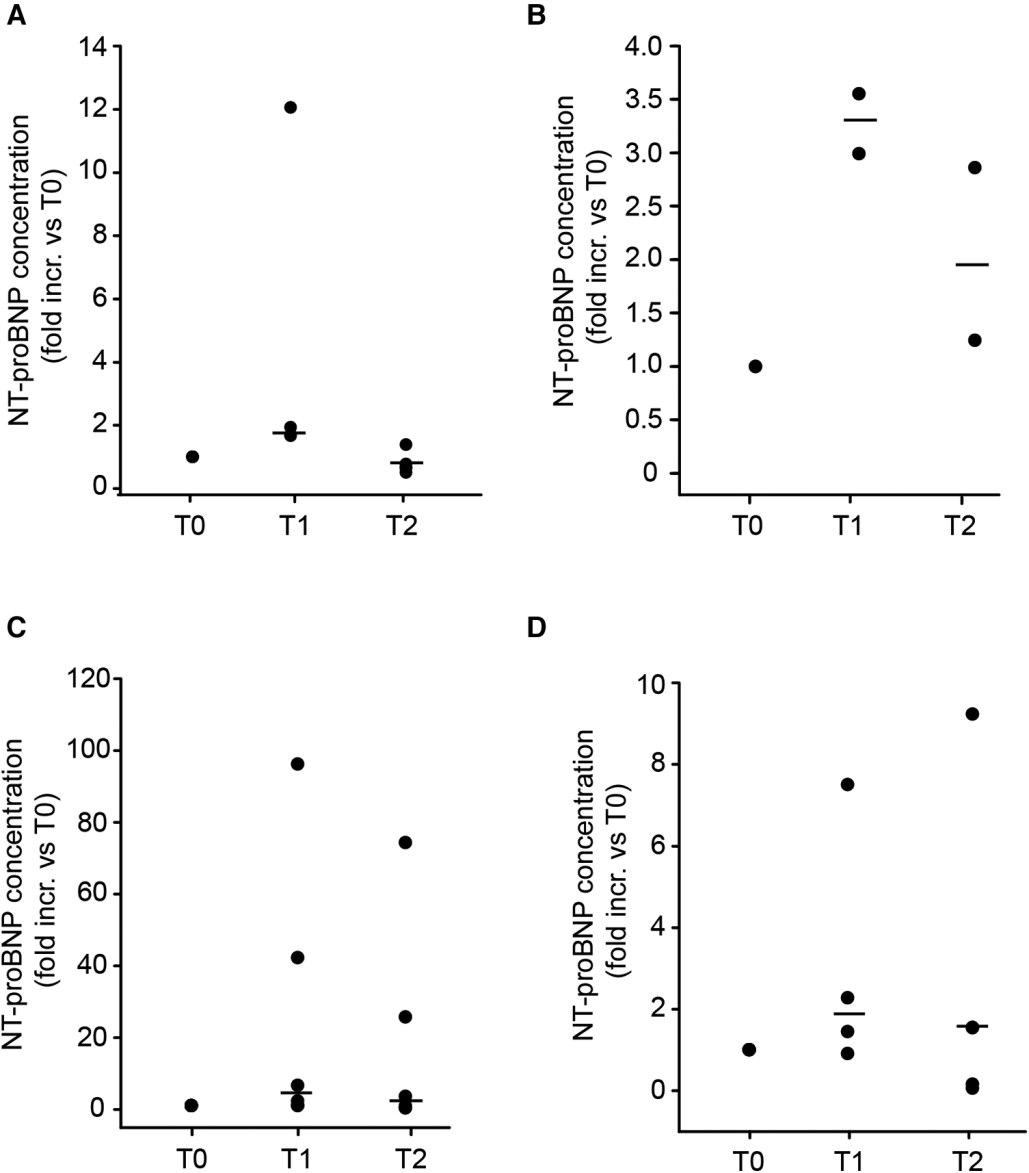
NT-proBNP concentrations of cardiac surgery patients with CPB. NT-proBNP was measured in the same samples from patients receiving levosimendan after induction of anaesthesia at dosages of (**A**) 1.25 mg of levosimendan (*n* = 5); (**B**) 1.25 mg levosimendan and a second dose (1.25 mg) 3–4 h after surgery (*n* = 2); (**C**) 2.5 mg levosimendan (*n* = 6); (**D**) 2.5 mg levosimendan and a second dose (2.50 mg) 5–21 h after surgery (*n* = 5). T0: before cardiac surgery; T1: shortly after cardiac surgery; T2: First day after cardiac surgery. Data are expressed as dot plots with the respective median (black line). No significant difference between the different time points within each treatment group was found (one-way ANOVA).

NT-proBNP (N-terminal pro B-type natriuretic peptide) is routinely used to aid diagnosis of heart failure, predict outcomes, and to monitor therapeutic effects (25). As levosimendan has been shown to reduce NT-proBNP levels (26), it may be used as a surrogate marker for detection of the therapeutic effect of levosimendan. The NT-proBNP levels in patients with CPB were measured at T0 (before surgery), T1 and T2, respectively, and set in relation to every patient´s value at T0. As seen in Supplementary Table S2, NT-proBNP concentrations of all patients were over the normal range (755 ± 156 pg/ml) before surgery, which is in accordance with their cardiac insufficiency. The NT-proBNP values increased after the surgery at T1 (1,678 ± 210.3 pg/ml; Figure 2). Although the NT-proBNP levels declined the day after surgery (T2: 999.8 ± 193.6 pg/ml), no significant reduction of the NT-proBNP level compared to T0 was observed with any of the applied dose regimens (Figures 2A–D).

## 4 Discussion

Levosimendan is used in patients with reduced EF due to its positive inotropic effect. While meta-analysis of observational studies showed positive effects on morbidity and mortality of cardiac surgery patients (10–12), the results from randomized controlled trials failed to reach statistical significance regarding these endpoints (13–15). Of note, none of these studies assessed serum concentrations of levosimendan, OR-1855 and OR-1896. Moreover, patient specific factors influencing for instance hemodynamics to variable degrees may also influence treatment responses of levosimendan. Therefore, an individualized therapeutic approach may be needed to ensure an adequate therapeutic response. In our center, the levosimendan dose is adjusted individually to fit patientś specific needs to become hemodynamically stable (16). Although a retrospective study, analysing the effects of this individualized therapeutic regimen, showed positive clinical outcomes when comparing this patient population with published studies using higher levosimendan doses, it was not evaluated if the observed outcomes were statistically improved (16). Therefore, the detection of serum levels of levosimendan and its metabolites in these patients would be of interest to confirm the observed effects. Furthermore, as the use of a CPB may change the pharmacokinetics of administered drugs (19, 27), this may in addition influence levosimendan serum levels. Therefore, in our opinion, therapeutic drug monitoring (TDM) of levosimendan and its metabolites is necessary in cardiac surgery patients with CPB to assess its therapeutic effect, especially in the case of individualized dosing. Thus, after establishing a new rapid LC-ESI-MS/MS-method for measuring serum concentrations of levosimendan and its metabolites simultaneously (21), we used this protocol to detect these compounds in serum from cardiac surgery patients with reduced EF, receiving levosimendan within the perioperative setting according to the individualized treatment approach reported by Woehrle et al. (16). We present, to the best of our knowledge, the first real-life study measuring both total and free unbound serum concentrations of levosimendan, OR-1896 and OR-1855 in cardiac surgery patients.

We detected concentrations of levosimendan ranging from below LLOQ to low levels directly after surgery (T1) after infusion of 1.25 mg. Higher serum concentrations were only measurable with a levosimendan administration of 2.5 mg. No existing serum concentrations could be measured at T2, due to its short half-life of approximately 1 h (23). Only in patients receiving a second dose of 2.5 mg, levosimendan was quantifiable at T2. The pharmacologically active metabolite OR-1896 was detected at T1 and T2, confirming firstly a rapid metabolization of levosimendan and secondly a longer half-life of OR-1896. Other studies have shown an increase of the metabolites during several days (28) (29), with mean peak concentrations after 6 days, suggesting that the OR-1896 concentrations measured in our study may further increase after surgery. Interestingly, even at the higher dose of 2.5 mg, almost no sample contained quantifiable amount of OR-1855, although OR-1896 was present. Reasons for this may be the rapid conversion to OR-1896. Furthermore, it remains unclear whether additional metabolic pathways exist other than in the intestinal tract, which was only shown in dogs (30). Moreover, administration of antibiotics can also alter the microbiome function (31) and may thus have an impact on the metabolism of levosimendan to OR-1855 in the intestine. Indeed, patients in our study received antibiotics (cefuroxime) prophylactically. Finally, differences in the measured serum concentrations of OR-1896 between fast and slow acetylators, due to different NAT2 genotypes, have been demonstrated (7) and may also contribute to lower serum levels of OR-1855. Due to the retrospective design of our study, we were unable to perform NAT2 genotyping.

Levosimendan is usually administered intravenously as a bolus injection of 6–12 µg/kg for 10 min followed by a continuous infusion over a period of 24 h at a dosage of 0.1 µg/kg/min (32–34). Cmax concentrations of 30.6 ± 2.2 ng/ml (after 18 h; normal acetylators), 37.5 ± 4.7 ng/ml (after 24 h; rapid acetylators) and 38.3 ± 3.6 ng/ml (after 18 h; slow acetylators) were reached in healthy men (7, 8, 35) after an infusion over 24 h. In randomized clinical trials with cardiac surgery patients, peak levosimendan concentrations of 98 ± 33 ng/ml were measured 2 h after the start of infusion (28) and 30 ng/ml 24 h after the start of infusion (bolus injection of 12 µg/kg followed by 0.2 µg/kg/min 24 h preoperatively) (29). In our study, patients received levosimendan doses of 1.25 mg or 2.5 mg according to an individualized therapeutic approach (17) with a flow rate between 0.2 and 0.25 μg kg^–1^·min^–1^ with the goal to prevent a postoperative LCO syndrome and concurrently avoid excessive vasodilation, which is a known side-effect especially during bolus administration. After surgery, the highest median concentrations were reached with a single dose of 2.5 mg levosimendan (10.4 ng/ml at T1) but were still considerably lower than the concentrations measured by Eriksson et al. (28) and Leppikangas et al. (29) as mentioned above. Even in patients receiving a second higher dose of 2.5 mg after surgery, only a levosimendan concentration of 3.9 ng/ml could be detected the day after surgery (T2). Hemodilution, which can occur upon CPB, may decrease circulating levels of levosimendan. However, the albumin concentrations in the patients of our study were within the normal range, suggesting that hemodilution did not occur. Moreover, the levels of OR-1855 and OR-1896 24 h after start of the levosimendan infusion in the study from Leppikangas et al. (29) were similar to the metabolite concentrations measured at T2 in our study. Yet, here, most of the samples showed OR-1855 concentrations below LLOQ. This discrepancy is probably due to the administered lower total levosimendan dose in our study. A direct comparison with clinical trials measuring serum or plasma levels of levosimendan is difficult, as available studies were performed in either healthy controls or in cardiac surgery patients (7, 8) with different dosing regimens (28, 29). Therefore, to be able to evaluate if the CPB procedure may have an impact on levosimendan and thus metabolite serum concentrations, we simulated the serum concentrations of levosimendan, which were to be expected after the respective dose in each patient. The estimated concentrations were significantly higher than the measured concentrations, regardless of treatment regimen. This may indicate, that the CPB procedure indeed may influence levosimendan drug levels in cardiac surgery patients. Moreover, both the measured and the simulated levosimendan concentrations showed a high variation between patients, suggesting that a rapid TDM in this setting may be advantageous.

Since the study of Woehrle et al. observed positive clinical effects of the individualized treatment approach (16), it is of interest to investigate if the detected concentrations are still high enough to induce a therapeutic response. Levosimendan has a high binding affinity to serum albumin with a pharmacologically active unbound fraction (UF) of only 1%–2%, whereas the UF of OR-1855 and OR-1896 is higher (approx. 60%) (23). Interestingly, we observed lower UF of levosimendan and both metabolites despite normal albumin concentrations. Of note, the majority of the samples had such low total serum concentrations of levosimendan that an UF of 1%–2%, as described in the literature, would be far below LLOQ. Moreover, several samples showed total concentration levels of levosimendan below LLOQ, suggesting a possible lack of therapeutic effects. As we measured few anonymized samples retrospectively from the local biobank, the left ventricular EF or the vasopressor demand after surgery for the respective patient could not be used to evaluate a therapeutic effect of levosimendan. However, the clinical data of our study population are shown within the study by Woehrle et al. (16). Here, we detected the cardiac marker NT-proBNP (36) in the patient samples, as levosimendan has been described to reduce NT-proBNP levels already after 24 h (37). Although we noticed a strong increase in NT-proBNP levels upon surgery, which is not surprising and has been shown before (38), and which declined at T2, no significant reduction of NT-proBNP levels between T0 and T2 were observed. Although levosimendan treatment did not improve NT-proBNP levels in comparison to baseline (before surgery), it still may reduce a further increase in NT-proBNP levels, which were shown to rise up to four days after surgery with CPB (39, 40). On the other hand, patients with levosimendan and metabolite levels below LLOQ did not show higher NT-proBNP levels compared to patients with levels over LLOQ (data not shown). Apart from the measurements of hemodynamic clinical outcomes, no specific biomarker detecting effects of levosimendan on cardiac function exist. Although NT-proBNP is a reliable marker for cardiac function *per se*, it is influenced by invasive surgery and its use in this setting is therefore limited. The matrix metalloproteinase (MMP) −2 is another biomarker for cardiac function, which decreases upon levosimendan treatment (41). MMP-2 is however influenced by CPB (42) and therefore not suitable in this setting. Suitable biomarkers for levosimendan needs to be further investigated.

Due to the retrospective design of our study with a limited number of available samples, there are several limitations needing consideration when evaluating the obtained data. First, as samples from later time points after the first day of surgery were not available, it is difficult to investigate the full pharmacokinetics of levosimendan and the metabolites in these patients. A longer duration of sample collection postoperatively may also enable a more rigid analysis of CPB as an influencing factor. Second, as we do not have access to the patient history or further patient data, such as comorbidities and comedications, due to the anonymization of samples, we cannot exclude all possible confounding parameters. Third, it is challenging to evaluate the therapeutic effects of levosimendan in a real-life patient population. Due to the anonymization, we could not use EF progress or other hemodynamic parameters to evaluate this. Moreover, cardiac biomarkers may give an indication to the effectiveness but are at the same time influenced by the invasive cardiac surgery, limiting their validity.

Although the investigation shows limitations, it should be noted that in most patients receiving an individualized treatment regimen, levels of levosimendan, OR-1855 and OR-1896 were low. Positive hemodynamic effects in patients have been shown with levosimendan plasma concentrations of as low as 15 ng/ml (43). Considering that we measured lower serum concentrations in 14 of 18 patients and that over half of the levels were even below LLOQ, the data questions a therapeutic effect on cardiac function in these patients. The extremely low unbound fractions of levosimendan, considering most levels being even below LLOQ, strengthen this note. Moreover, we observed high variations in serum levels between patients receiving the same dose. We therefore strongly recommend performing rapid TDM in patients receiving the individualized levosimendan treatment. Moreover, we demonstrate the need for further larger prospective studies with longer follow-up periods, even testing different timing and duration of levosimendan application, investigating both serum concentrations of levosimendan and metabolites and clinical outcomes combined with different cardiac biomarkers to investigate the therapeutic effect of levosimendan. This may help in optimizing the individualized levosimendan treatment regimen and in developing future guidelines for this critical patient population.

## Data Availability

The original contributions presented in the study are included in the article/Supplementary Material, further inquiries can be directed to the corresponding author.
