# Supplementary material for: Serum concentrations of levosimendan and its metabolites OR-1855 and OR-1896 in cardiac surgery patients with cardiopulmonary bypass
Source: Front Cardiovasc Med. 2024 Aug 8;11:1406338. doi: 10.3389/fcvm.2024.1406338 (PMC11338783; doi:10.3389/fcvm.2024.1406338)
Supplement: Supplementary file 1 [file Datasheet1.pdf]

Supplementary tables

S Table 1

Patient	Levosimendan (mg)		Levosimendan TSC (ng/ml)		OR-1855 TSC (ng/ml)		OR-1896 TSC (ng/ml)	
	S	P.S	T1	T2	T1	T2	T1	T2
1	1.25	-	bLLOQ	bLLOQ	bLLOQ	bLLOQ	bLLOQ	1.5
2	1.25	-	40.6	bLLOQ	bLLOQ	bLLOQ	bLLOQ	0.7
3	1.25	-	2.5	bLLOQ	0.4	0.7	bLLOQ	bLLOQ
4	1.25	-	1.4	bLLOQ	i.s.v	i.s.v	i.s.v	i.s.v
5	1.25	-	0.4	bLLOQ	bLLOQ	bLLOQ	0.9	bLLOQ
6	1.25	1.25	1.1	bLLOQ	2.8	bLLOQ	bLLOQ	0.8
7	1.25	1.25	0.2	bLLOQ	i.s.v	i.s.v	i.s.v	i.s.v
8	2.5	-	2.2	bLLOQ	bLLOQ	bLLOQ	5.1	4.0
9	2.5	-	11.2	bLLOQ	bLLOQ	bLLOQ	1.4	0.7
10	2.5	-	9.6	bLLOQ	bLLOQ	bLLOQ	bLLOQ	4.0
11	2.5	-	1.9	bLLOQ	bLLOQ	bLLOQ	bLLOQ	0.6
12	2.5	-	28.3	bLLOQ	bLLOQ	bLLOQ	bLLOQ	1.6
13	2.5	-	13.2	bLLOQ	i.s.v	i.s.v	i.s.v	i.s.v
14	2.5	2.5	1.8	bLLOQ	bLLOQ	bLLOQ	2.0	4.1
15	2.5	2.5	i.s.v.	0.2	i.s.v	1.0	i.s.v	0.6
16	2.5	2.5	8.6	13.7	bLLOQ	bLLOQ	bLLOQ	0.4
17	2.5	2.5	26.1	0.5	bLLOQ	bLLOQ	bLLOQ	0.6
18	2.5	2.5	1.9	7.3	bLLOQ	bLLOQ	bLLOQ	bLLOQ

Supplementary Table 1. Applied levosimendan doses and measured total serum concentrations (TSC) of levosimendan, OR-1855 and OR-1896

Column two shows the total applied levosimendan dose(s) in mg; Column three – five shows the respective measured total serum concentration (TSC) of levosimendan, OR-1896 and OR-1855 in ng/ml. T1: Shortly after surgery; T2: First day after surgery. S: Surgery (application after anesthesia); P.S: Post-surgery (application on first post-operative day); bLLOQ: below lower limit of quantification; i.s.v: insufficient serum volume.

S Table 2

Patient	T0 (pg/mL)	T1 (pg/mL)	T2 (pg/mL)
1	70.2	846.9	45.0
2	1303.0	2165.1	1810.1
3	651.8	1261.1	327.7
4	1673.4	2833.3	1137.0
5	877.3	i.s.v	675.3
6	409.8	1456.3	510.1
7	998.2	2986.9	2855.4
8	921.9	2130.7	374.2
9	293.7	1981.4	1070.0
10	49.0	2073.2	1259.9
11	12.2	1177.0	909.1
12	2484.9	2752.4	2478.7
13	735.5	806.5	327.3
14	659.3	602.2	37.0
15	72.0	540.0	664.4
16	945.9	i.s.v	1468.0
17	641.1	931.2	101.4
18	681.8	1555.4	1048.1

Supplementary Table 2. NT-proBNP concentrations of all patient samples

T0: before surgery; T1: At the end of surgery; T2: First day after surgery. Patient 1-5: Levosimendan (1.25 mg) application started after induction of anesthesia. Patient 6-7: First levosimendan (1.25 mg) application started after induction of anesthesia and the second application (1.25 mg) started after surgery. Patient 8-13: Levosimendan (2.5 mg) application started after induction of anesthesia. Patient 14-18: First levosimendan (2.5 mg) application started after induction of anesthesia and the second application (2.5 mg) started after surgery.
